# A novel polyketide synthase gene cluster in the plant pathogenic fungus *Pseudocercospora fijiensis*

**DOI:** 10.1371/journal.pone.0212229

**Published:** 2019-02-08

**Authors:** Roslyn D. Noar, Elizabeth Thomas, Margaret E. Daub

**Affiliations:** 1 Department of Plant Pathology, North Carolina State University, Raleigh, NC, United States of America; 2 Department of Plant and Microbial Biology, North Carolina State University, Raleigh, NC, United States of America; Universite Paris-Sud, FRANCE

## Abstract

*Pseudocercospora fijiensis*, causal agent of black Sigatoka of banana, produces polyketide synthase (PKS) pathways shown to be important in disease development by related Dothideomycete fungi. Genome analysis of the *P*. *fijiensis PKS8-1* gene identified it as part of a gene cluster including genes encoding two transcription factors, a regulatory protein, a glyoxylase/beta-lactamase-like protein, an MFS transporter, a cytochrome P450, two aldo/keto reductases, a dehydrogenase, and a decarboxylase. Genome analysis of the related pathogens *Pseudocercospora musae*, *Pseudocercospora eumusae*, and *Pseudocercospora pini-densiflorae*, identified orthologous clusters containing a nearly identical combination of genes. Phylogenetic analysis of PKS8-1 identified homology to PKS proteins in the monodictyphenone and cladofulvin pathways in *Aspergillus nidulans* and *Cladosporium fulvum*, respectively. Analysis of clustered genes showed that the *PKS8-1* cluster shares genes for enzymes involved in the production of the emodin intermediate in the monodictyphenone and cladofulvin pathways, but differs in many genes, suggesting production of a different metabolic product. Time course analysis of gene expression in infected banana showed up-regulation of *PKS8-1* and four of eight clustered genes as early as 2 weeks post-inoculation and remaining high through 9 weeks. Overexpression of the pathway through constitutive expression of an *aflR-*like transcription factor gene in the cluster resulted in increased expression in culture of *PKS8-1* as well as the four clustered genes that are up-regulated in infected plants. No differences were seen in timing or severity of disease symptoms with the overexpression strains relative to controls, however gene expression analysis showed no difference in expression *in planta* by an overexpression strain relative to controls. Thus constitutive expression of the *aflR*-like gene is not sufficient to upregulate the pathway above normal expression *in planta*. Pathway expression during all phases of disease development and conservation of the pathway in related *Pseudocercospora* species support a role for this pathway in disease.

## Introduction

Black Sigatoka disease, caused by the fungus *Pseudocercospora fijiensis*, is considered the most economically damaging disease of banana and plantain. Fungicide sprays accounting for 25–30% of the total banana production cost are currently the primary means of control for this disease, and without them, 50% yield loss can result [1–5]. The disease causes loss of photosynthetic capacity due to necrotic streaking on the banana leaves, as well as premature fruit ripening. The importance of this disease has led to extensive research efforts to understand the disease process, including sequencing of both the *P*. *fijiensis* and banana genomes [6–9]. Significant recent work has focused on defense responses, comparing disease development in susceptible vs resistant banana genotypes [6, 10–12], on characterization of effectors produced by *P*. *fijiensis* [13–15], and on the role of osmotic stress and signaling pathways in pathogenicity [16, 17]. Recent studies have also focused on secondary metabolic pathways in the fungus that may serve as pathogenicity mechanisms [7, 18–20].

Both conidia and ascospores of *P*. *fijiensis* can germinate on the banana leaf, growing epiphytically before entering the leaf via the stomata [2]. The hyphae encircle the substomatal cavity and grow intercellularly in a biotrophic phase, before switching to a necrotrophic lifestyle and causing the characteristic necrotic leaf streaks. In related fungi, secretion of polyketide toxins has been shown to facilitate necrotrophic growth. For example, *Cercospora* spp. and *Ramularia collo-cygni* produce the perylenequinone cercosporin and the anthraquinone rubellin, respectively, through polyketide biosynthetic pathways. These are both light-activated toxins that produce reactive oxygen species that kill the host tissue [21, 22]. It has long been suspected that *P*. *fijiensis* and other related Sigatoka banana pathogens produce a polyketide toxin with a similar mode of action, because Sigatoka disease symptoms are less severe in partial shade [2, 4]. For example, an early method of managing symptoms of yellow Sigatoka was to grow plants in the shade of coffee trees, which increased marketable banana yields by 50% [23, 24]. More recent studies have confirmed reduced incidence of black Sigatoka under shaded agroforestry systems as well as under high-density cultivation where plants are shaded [25, 26].

Previous studies have identified several polyketide toxins from *P*. *fijiensis*. These include juglone, 4-hydroxyscytalone, and 2,4,8-trihydroxytetralone (2,4,8-THT), all of which are shunt pathway metabolites of melanin biosynthesis [27, 28]. Juglone was of particular interest as an important pathogenicity factor because it has light-dependent toxicity through inhibition of electron transport in banana chloroplasts [29], a finding that could explain the light-dependence observed for black Sigatoka symptoms. In addition, studies suggested that 2,4,8-THT is important for pathogenicity because host selectivity was observed when comparing its phytotoxic effects on resistant and susceptible banana cultivars [27]. Further, application of the fungicide tricyclazole, which blocks melanin biosynthesis and causes an accumulation of 2,4,8-THT, increased symptom development in *P*. *fijiensis*-infected banana plants [30]. Despite the early indication that melanin shunt pathway metabolites could play an important role in black Sigatoka disease symptoms, more recently it has been reported that disruption of the melanin-producing polyketide synthase has no effect on disease symptoms in *P*. *fijiensis* [2].

Polyketides play important roles for fungal pathogens beyond acting as phytotoxins, including roles as effectors [31] and as mycotoxins [32]. Genome sequencing of *P*. *fijiensis* and its close relatives *Pseudocercospora musae* and *Pseudocercospora eumusae* has enabled bioinformatics analyses of polyketide biosynthetic genes in these species [7, 18, 33]. Polyketides are synthesized by large, multi-domain polyketide synthase (PKS) enzymes [34]. The polyketide is then modified by other enzymes that are typically encoded together in gene clusters with the polyketide synthase gene in fungal genomes [35]. We previously used bioinformatics to predict seven polyketide synthase genes (*PKS2-1*, *PKS7-1*, *PKS8-1*, *PKS8-2*, *PKS8-4*, *PKS10-1*, and *PKS10-2*) and one hybrid PKS/NRPS gene (*Hybrid8-3*) in the *P*. *fijiensis* genome, along with their putative gene clusters [18]. Phylogenetic analysis showed that PKS10-1 formed a clade with PKS enzymes involved in melanin biosynthesis. Other *P*. *fijiensis* PKS enzymes were found in clades for known polyketides including fumonisin, produced by *Fusarium verticillioides* (PKS8-2), solanapyrone and alternapyrone, produced by *Alternaria solani* (PKS10-2 and 2–1, respectively), and lovastatin, produced by *Aspergillus terreus* (PKS8-4) [18]. Phylogenetic analysis did not identify close homology of PKS7-1 to any other PKS. RNA-Seq analysis showed that genes in the PKS7-1, 8–2, and 10–2 clusters were up-regulated in infected leaves as compared to culture, but the PKS2-1 and 10–1 cluster genes were more highly expressed in culture [18].

*PKS8-1* is one of the seven *P*. *fijiensis* genome PKS genes identified through our previous bioinformatics analysis [18]. The PKS8-1 enzyme was shown to have ketosynthase, acyltransferase, and acyl carrier protein domains, and the gene was clustered in the genome with a large number of genes common in secondary metabolite pathways. Phylogenetic analysis, however, did not identify any known homologs, and RT-PCR analysis showed greater expression in culture than in infected leaves. Here we present a more extensive analysis of *PKS8-1* and its associated cluster, including its relationship to PKS clusters involved in cladofulvin and monodictyphenone synthesis, a time course of expression of cluster genes during infection, and the impact on disease by overexpression of the cluster transcription factor.

## Results

### Comparison of the *PKS8-1* cluster with other Dothideomycete clusters

**Fig 1:**
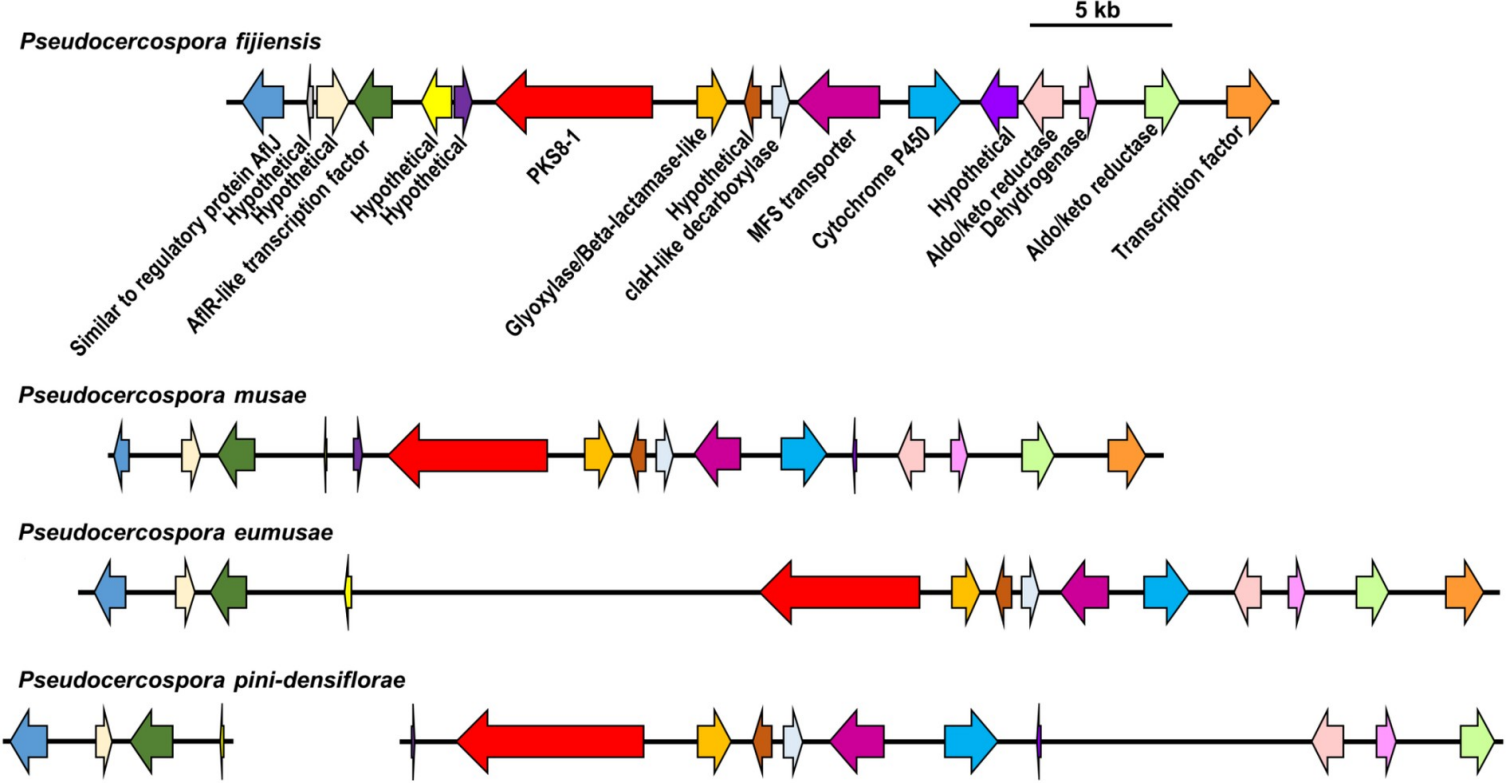
Comparison of *PKS8-1* gene clusters across different species. For *P*. *fijiensis*, *P*. *musae*, *P*. *eumusae*, and *P*. *pini-densiflorae*, *PKS8-1* and adjacent genes in the genome or their putative orthologs are shown by arrows indicating their direction. Putative orthologous genes are shown with the same color of arrow. A description of the putative function of each corresponding protein is shown under the *P*. *fijiensis* arrows. Scale bar indicates 5 kb.

In our previous study we identified *PKS8-1* as one of seven PKS genes in the *P*. *fijiensis* genome. *PKS8-1* was found to be clustered in the genome with genes commonly found in secondary metabolite clusters including two transcription factor genes and genes encoding a glyoxylase/beta-lactamase-like protein, an MFS transporter, a cytochrome P450, two aldo/keto reductases, a dehydrogenase, and a regulatory protein similar to AflJ from the aflatoxin biosynthesis pathway in *Aspergillus* [18]. In our previous analysis, we identified homologs of genes in the *PKS8-1* cluster from other Dothideomycetes, but did not determine whether these homologs are part of similar gene clusters. Therefore, we conducted tblastn searches for each predicted protein within the *P*. *fijiensis* gene cluster, using the set of Dothideomycete genomes listed previously [18], and also determined the location of each homolog within the genome scaffolds. This analysis confirmed that the *PKS8-1* gene cluster is conserved across many of the Dothideomycete species (S1 Table). Although not identified in our previous analysis due to the cutoff by bitscore of the top 10 PKS8-1 homologs [18], the genomes of the closely related species *P*. *musae*, *P*. *eumusae*, and *Pseudocercospora pini-densiflorae* also have orthologous gene clusters (Fig 1, S1 Table) containing a nearly identical combination of genes adjacent to the polyketide synthase. These include genes homologous to those encoding the AflR transcription factor and the AflJ regulatory protein in *Aspergillus*, a glyoxylase/beta-lactamase, an MFS transporter, a cytochrome P450, two aldo/keto reductases, an ethD domain-containing decarboxylase, and a dehydrogenase. The extremely similar gene clusters between *Pseudocercospora* species suggest that these species produce a similar polyketide.

### Comparison to cladofulvin and monodictyphenone-producing enzymes

**Fig 2:**
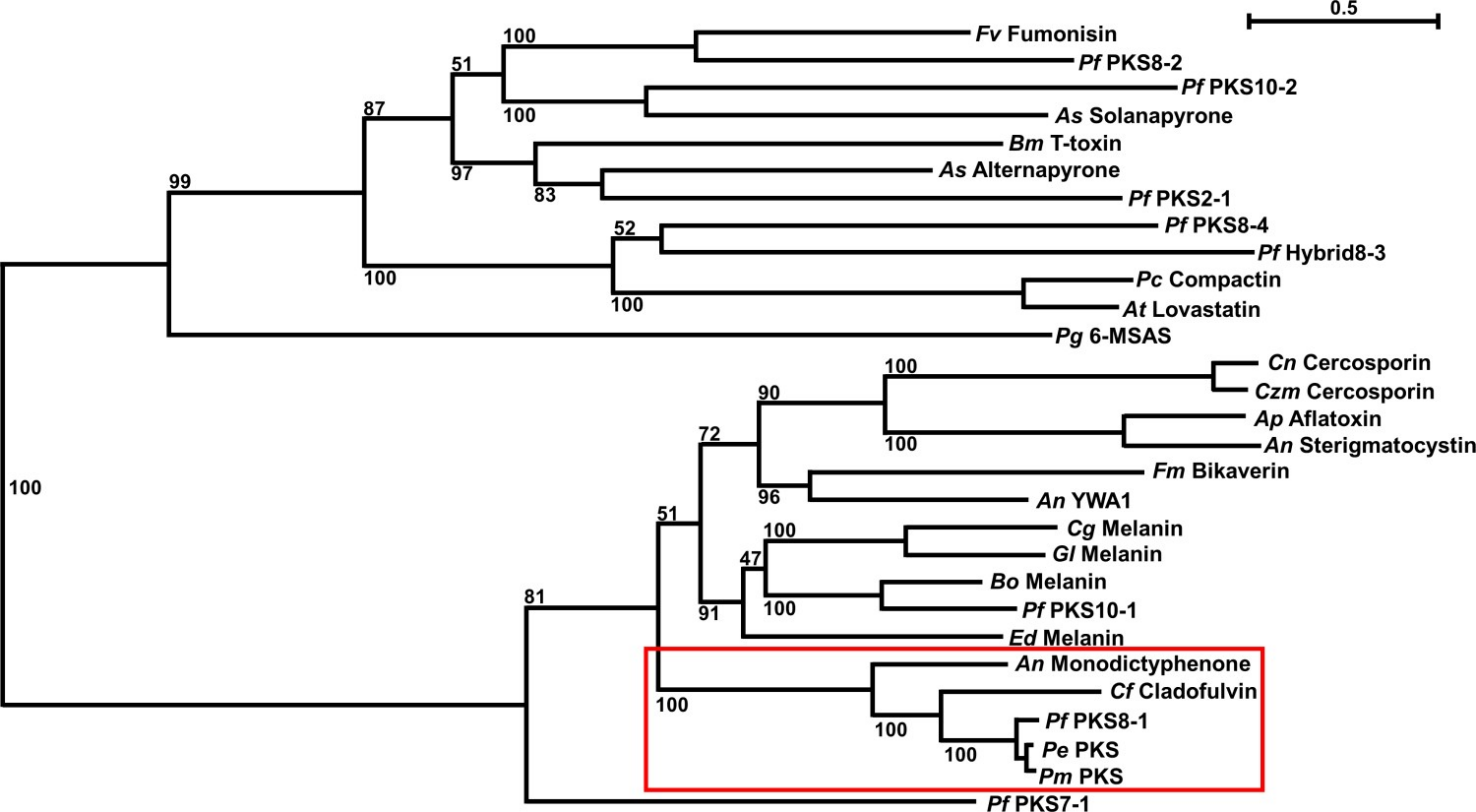
Phylogenetic analysis of PKS8-1. RAxML was used to create a maximum likelihood phylogenetic tree of PKS protein sequences, using the PKS sequences described previously [18] and others including the monodictyphenone-producing MdpG from *A*. *nidulans*, the cladofulvin-producing ClaG from *C*. *fulvum*, and the PKS8-1 homolog sequences identified from *P*. *musae* and *P*. *eumusae*. The clade containing PKS8-1, its *P*. *musae* and *P*. *eumusae* homologs, the monodictyphenone-producing PKS from *A*. *nidulans*, and the cladofulvin-producing PKS from *C*. *fulvum* is shown with a red box. Bootstrap values are indicated on the tree, and the scale bar indicates substitutions per site. *An* = *Aspergillus nidulans*; *Ap* = *Aspergillus parasiticus*; *As* = *Alternaria solani*; *At* = *Aspergillus terreus*; *Bm* = *Bipolaris maydis*; *Bo* = *Bipolaris oryzae*; *Cf* = *Cladosporium fulvum*; *Cg* = *Colletotrichum graminicola*; *Cn* = *Cercospora nicotianae*; *Czm* = *Cercospora zeae-maydis*; *Ed* = *Exophiala dermatitidis*; *Fm* = *Fusarium moniliforme*; *Fv* = *Fusarium verticillioides*; *Gl* = *Glarea lozoyensis*; *Pc* = *Penicillium citrinum*; *Pg* = *Penicillium griseofulvum*; *Pe* = *Pseudocercospora eumusae*; *Pf* = *Pseudocercospora fijiensis*; *Pm* = *Pseudocercospora musae*.

To predict the type of polyketide that may be produced by PKS8-1, we previously created a phylogenetic tree with PKS protein sequences from *P*. *fijiensis* and well-characterized PKS sequences from other species [18]. Our phylogenetic analysis showed that the PKS8-1 sequence was part of a clade of non-reducing PKS enzymes, but this analysis did not identify close, well-characterized homologs to PKS8-1. A separate analysis was done by Chang et al [7], who reported that there are three non-reducing PKS enzymes encoded by the *P*. *fijiensis* genome, consistent with our prediction that PKS7-1, PKS8-1, and PKS10-1 are non-reducing PKS enzymes in this fungus [7, 18]. Based on the ketosynthase (KS) and acyltransferase (AT) domains of these PKS enzymes, one non-reducing PKS from *P*. *fijiensis* was similar to those producing 1,8-dihydroxynaphthalene (DHN)-melanin, and another had similarity to PKS enzymes producing endocrocin and monodictyphenone [7]. The third non-reducing PKS enzyme did not have similarity to other PKS sequences [7]. Furthermore, the Dothideomycete *Cladosporium fulvum* has been shown to produce the anthraquinone cladofulvin via a PKS and gene cluster homologous to the monodictyphenone-producing gene cluster in *Aspergillus nidulans* [36, 37]. To determine whether the predictions of Chang et al would be the same using the entire PKS sequence for analysis, and to characterize the phylogenetic relationship between the monodictyphenone and cladofulvin-producing sequences with PKS8-1, we added sequences for the endocrocin and monodictyphenone-producing MdpG from *A*. *nidulans* and the cladofulvin-producing ClaG from *C*. *fulvum* to PKS sequences previously analyzed from *P*. *fijiensis* and other fungi [18], and created a new maximum likelihood phylogenetic tree (Fig 2). This analysis confirmed that whether the entire PKS sequence or only the KS and AT domains are used to create a phylogenetic tree, PKS8-1 and its homologs in other *Pseudocercospora* species form a clade with a strong bootstrap value with the monodictyphenone-producing MdpG PKS from *A*. *nidulans* as well as the cladofulvin-producing ClaG PKS from *C*. *fulvum* (Fig 2). Domain analysis indicates that PKS8-1, MdpG, and ClaG all share the same catalytic domain organization, with starter unit acyltransferase (SAT), ketosynthase (KS), acyltransferase (AT), product template (PT), and acyl carrier protein (ACP) domains [18, 37, 38]. These domains are indicative of non-reducing PKS proteins.

**Fig 3:**
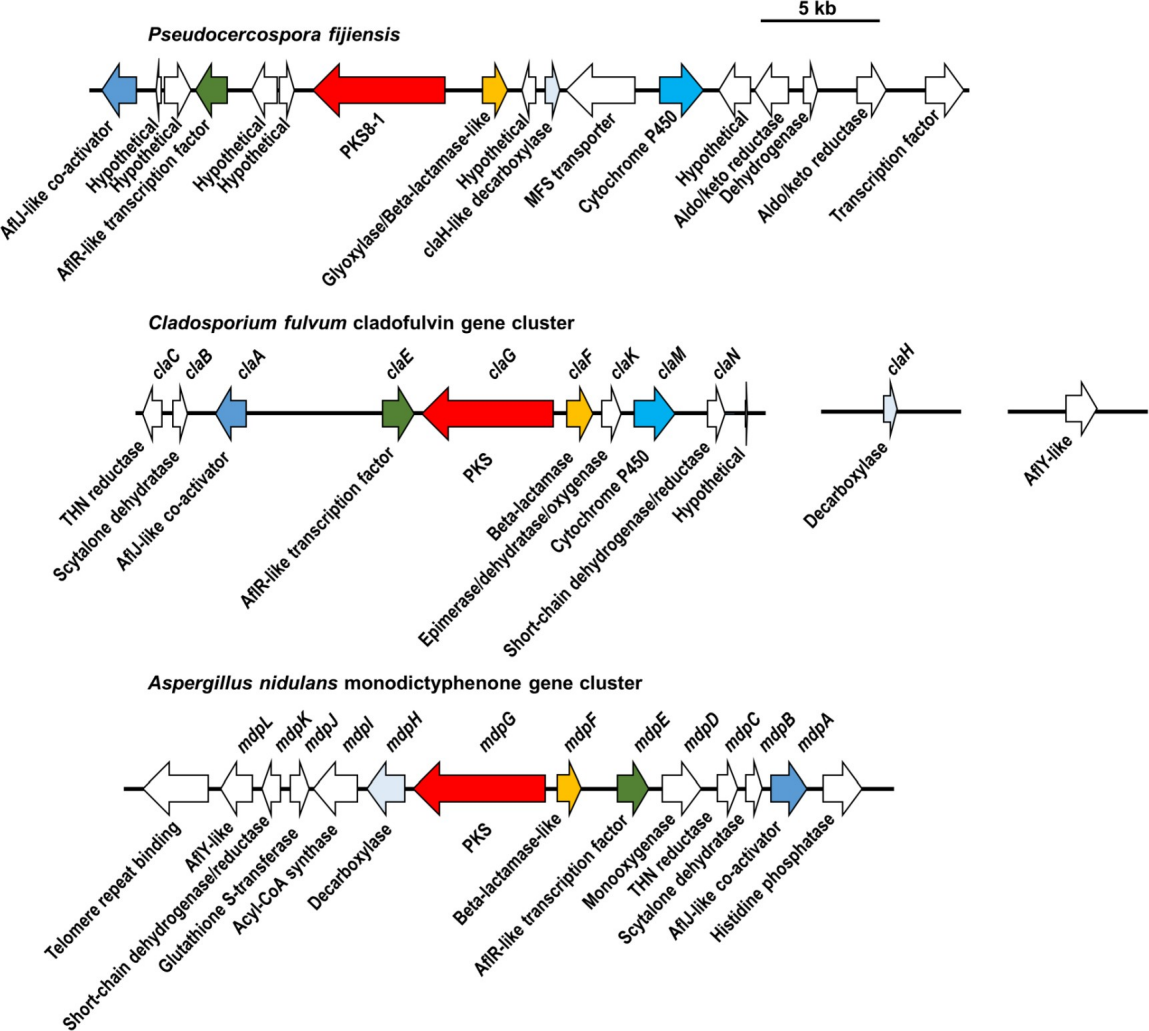
Comparison of *P*. *fijiensis PKS8-1* cluster, the cladofulvin-producing cluster from *C*. *fulvum*, and monodictyphenone-producing cluster from *A*. *nidulans*. For the *PKS8-1* cluster, the cladofulvin-producing cluster from *C*. *fulvum*, and the monodictyphenone-producing cluster from *A*. *nidulans*, each gene is shown in the genome with an arrow indicating its direction. Gene names, where applicable, are shown above the arrows, and descriptions of the putative function of each gene are shown below the arrows. Putative orthologs are shown with the same color arrow, and other genes are shown with white arrows. Details on cluster gene comparisons are shown in S2 Table.

Since the phylogenetic analysis in Fig 2 showed that PKS8-1, the monodictyphenone-producing MdpG, and the cladofulvin-producing ClaG form a clade, the gene clusters for each PKS were compared. Blast searches were used to identify putative orthologs in the *P*. *fijiensis* genome for each gene in the monodictyphenone-producing gene cluster from *A*. *nidulans* [38] and the cladofulvin-producing gene cluster from *C*. *fulvum* [37], and to determine whether these putative orthologs flank the *PKS8-1* gene in the *P*. *fijiensis* genome (Fig 3, S2 Table). This analysis revealed that all three clusters share genes encoding a PKS, a beta-lactamase-like enzyme, an AflR-like transcription factor, an AflJ-like regulatory protein, and an ethD domain-containing decarboxylase homologous to MdpH from *A*. *nidulans* and ClaH from *C*. *fulvum* (Fig 3). The *PKS8-1* gene cluster and the cladofulvin-producing gene cluster also share a gene encoding a cytochrome P450 (Fig 3, S2 Table). However, there are also differences between the clusters. The putative *PKS8-1* cluster from *P*. *fijiensis* contains genes encoding an MFS transporter, two aldo/keto reductases, a dehydrogenase, a transcription factor, and six hypothetical genes that the *A*. *nidulans* and *C*. *fulvum* clusters lack (Fig 3). Conversely, the *A*. *nidulans* cluster [38] encodes a short-chain dehydrogenase, a glutathione S-transferase, an acyl-CoA synthase, a monooxygenase, a reductase, a dehydrogenase, and an AflY-like hypothetical protein that the *P*. *fijiensis* cluster lacks (Fig 3). The *C*. *fulvum* cluster [37] encodes a trihydroxynaphthalene reductase, a scytalone dehydratase, an epimerase/dehydratase/oxygenase, a short-chain dehydrogenase, and an AflY-like hypothetical protein that the *P*. *fijiensis* cluster lacks (Fig 3). These results suggest that the two clusters likely encode similar but different polyketide products.

### RNA-Seq analysis of *PKS8-1* gene cluster

**Fig 4:**
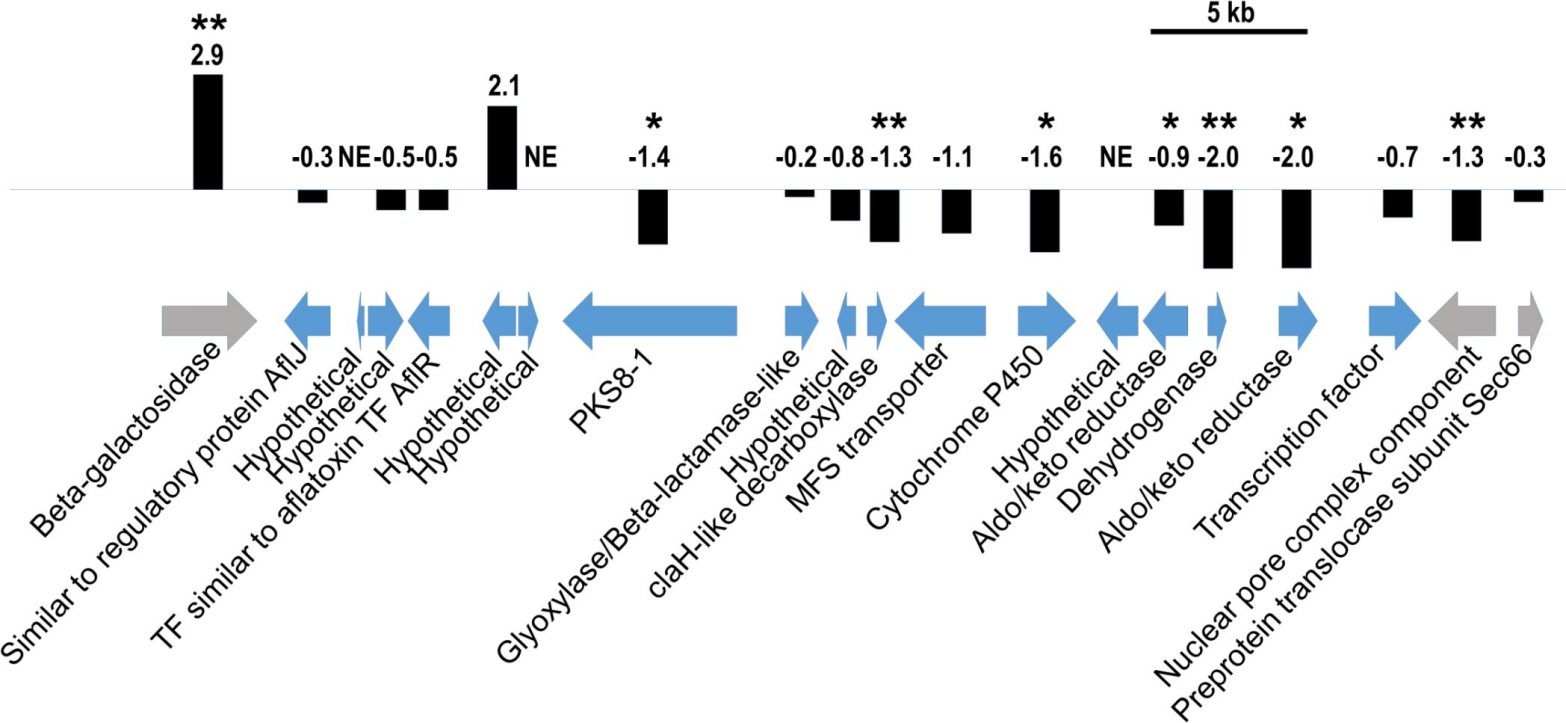
RNA-Seq analysis of *PKS8-1* cluster genes. *PKS8-1* and adjacent genes in the *P*. *fijiensis* genome are represented by arrows indicating their direction, with descriptions of putative protein functions indicated below the arrows. Blue arrows = genes predicted by Noar and Daub [18] to be part of the *PKS8-1* gene cluster; Gray arrows = genes predicted by Noar and Daub [18] to flank the *PKS8-1* gene cluster. Scale bar indicates 5 kb. Black bars are proportional to log2FC values of expression in leaf tissue vs. culture medium. Single asterisks represent significant expression differences at p<0.05, and double asterisks represent significant expression differences at p<0.01. NE = no expression detected.

Previously, we analyzed the expression pattern of *PKS8-1* in isolate 10CR1-24, in infected leaf tissue and in culture medium, using RT-PCR assays, and showed that *PKS8-1* is expressed under both conditions [18]. To learn more about the expression pattern of *PKS8-1* and the adjacent genes in its putative biosynthetic cluster, we used our RNA-Seq dataset [18, 19] to analyze the expression pattern of these genes in the infected leaf tissue versus in Potato Dextrose Broth (PDB) culture medium in isolate 14H1-11A, isolated from Honduras in 2014 [18] (Fig 4). Tissue samples were harvested from symptomatic leaves at 6 weeks post-inoculation at the initiation of the necrotrophic state. For *PKS8-1* expression, this analysis agreed with previous results in isolate 10CR1-24 using RT-PCR assays [18]. *PKS8-1* was expressed under both conditions, although it had slightly lower expression (log2FC = -1.4) in the infected leaf tissue compared to in PDB (Fig 4). Nearby genes encoding a cytochrome P450, two aldo/keto reductases, a dehydrogenase, and a ClaH-like decarboxylase also had slightly lower expression in infected leaf tissue (Fig 4). Other nearby genes had negative log2FC values, but these values were not significantly different (genes encoding AflJ-like and AflR-like proteins, beta-lactamase-like proteins, an MFS transporter, another transcription factor, and two hypothetical genes) (Fig 4). Because of the small expression changes, it was not possible to clarify the boundaries of the *PKS8-1* biosynthetic cluster using these data.

### Time course of expression of *PKS8-1* and clustered genes during disease development

**Fig 5:**
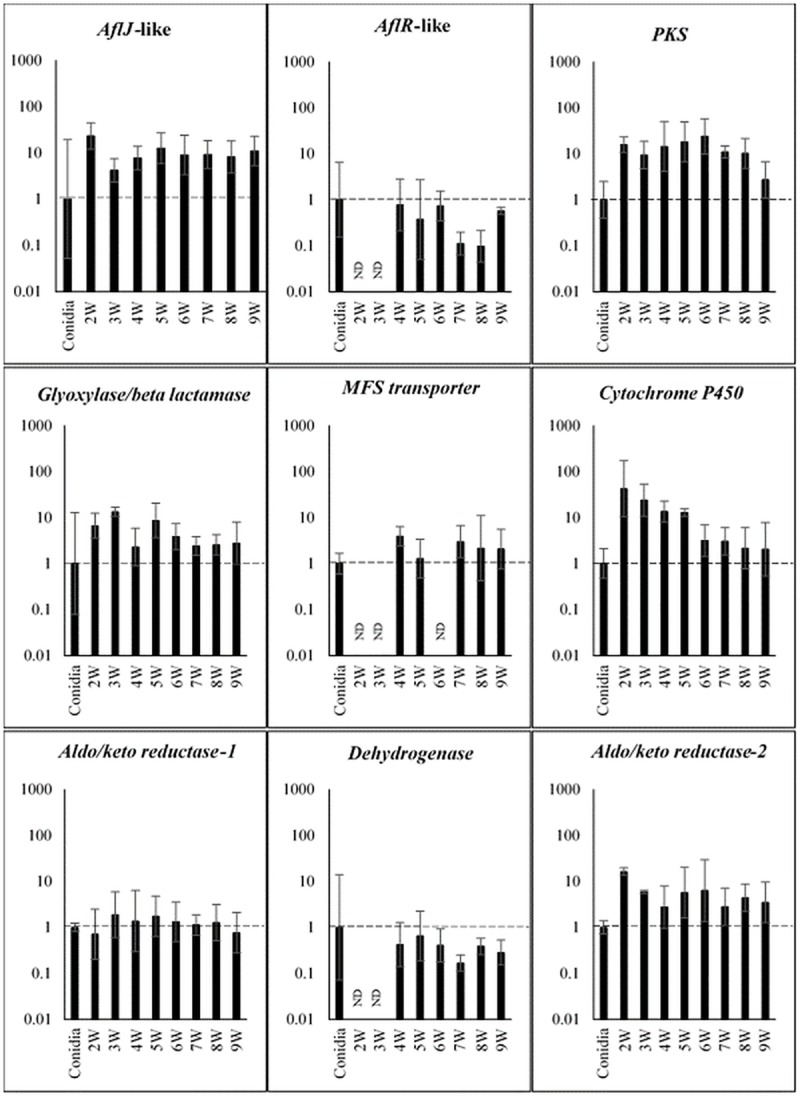
Time course of expression of *PKS8-1* and cluster genes in banana inoculated with wild-type *P*. *fijiensis* isolate 10CR1-24. Cluster genes assayed are those shown in Fig 4. Fold-change gene expression is shown relative to expression in conidia, set to 1. Tissue was harvested and assayed weekly starting at 2 weeks post-inoculation through 9 weeks. Results for each gene are shown as a bar graph in log scale, with error bars indicating standard error from three biological replicates. ND = not detected; W = week.

To better understand the possible role of *PKS8-1* and its clustered genes during infection and disease development, we conducted a time course experiment of expression in inoculated leaves relative to expression in germinating conidia. Plants were inoculated with wild-type *P*. *fijiensis* isolate 10CR1-24. Leaves from infected banana plants were harvested weekly for expression analysis using RT-qPCR. Results are shown in Fig 5. Relative to conidia, significantly increased expression was shown for *PKS8-1* as well as the clustered *aflJ*-like, glyoxylase/beta-lactamase, cytochrome P450, and the aldo/keto reductase gene furthest from *PKS8-1* (aldo/keto reductase-2). For all these genes, expression was detected as early as 2 weeks after inoculation, and with the exception of the cytochrome P450 gene, remained high through the full 9 weeks of the experiment. Expression of the cytochrome P450 gene was highest early in the disease cycle, and then decreased. By contrast, expression of genes for the AflR*-*like transcription factor, MFS transporter, the aldo/keto reductase located closest to the *PKS8-1* gene (aldo/keto reductase-1), and dehydrogenase was not increased (Fig 5).

### Overexpression of the *PKS8-1* gene cluster

To further characterize the role of the *PKS8-1* cluster, we conducted experiments to overexpress the cluster by constitutive expression of a transcription factor gene. There are two genes predicted to encode transcription factors with DNA-binding domains in the putative *PKS8-1* cluster (Fig 4). One transcription factor is predicted to be AflR-like and contains a GAL4-like Zn2Cys6 binuclear cluster DNA-binding domain. Overexpression of *aflR* has been successfully used to increase transcription of genes in the aflatoxin biosynthetic pathway in *Aspergillus flavus*, leading to increased aflatoxin production [39]. The other transcription factor encoded in the *PKS8-1* cluster contains a fungal-specific transcription factor domain and a fungal transcription factor regulatory middle homology region. Homologs to this regulator have not been characterized, however, and the gene is not present in the *P*. *pini-densiflora* cluster sequence. Although our expression analysis did not show increased expression of the *aflR*-like gene in infected leaves compared to conidia, the successful use of *aflR* overexpression in up-regulating the entire aflatoxin pathway in *A*. *flavus* led us to test if overexpression of the *aflR-*like gene could upregulate the PKS8-1 pathway and give insight into its function.

**Fig 6:**
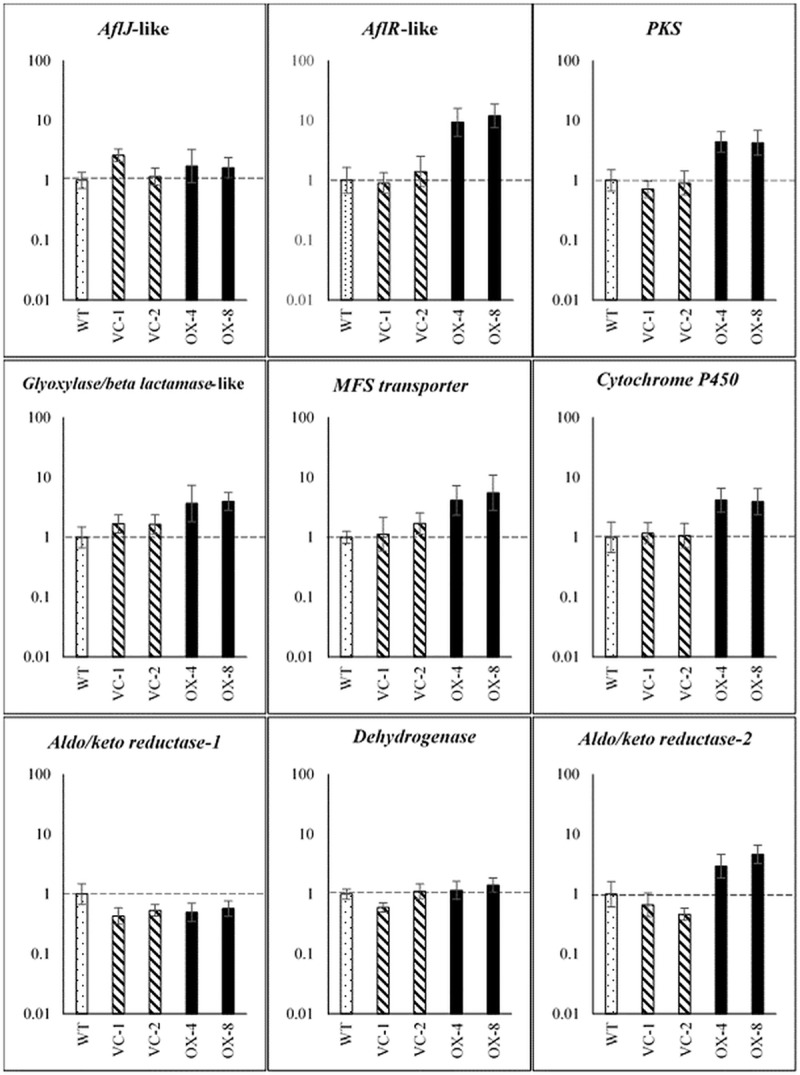
RT-qPCR assays of *PKS8-1* cluster genes in overexpression transformants of the *aflR*-like gene and vector controls relative to wild-type expression in culture. The wild type, two transformants of vector control (VC-1, VC-2), and two transformants of the *aflR*-like transcription factor overexpression (OX-4, OX-8) were grown in PDB medium. RT-qPCR assays were performed on these samples for the *PKS8-1* cluster genes. Fold-change gene expression is shown on a log scale relative to wild type set to 1. Error bars indicate standard error from six biological replicates. Dotted bar: wild type; hatched bars: vector controls; black bars: overexpression strains.

To create overexpression strains we transformed *P*. *fijiensis* isolate 10CR1-24 with the *PKS8-1* cluster *aflR*-like transcription factor gene under the control of the constitutive *Aspergillus nidulans trpC* promoter [40]. No phenotypic differences were noted in transformants of the transcription factor overexpression construct. They did not differ in colony appearance or growth rate on culture medium *in vitro* and were also not altered in conidia production or appearance. Transformants grown in PDB were tested by RT-qPCR for expression of *PKS8-1* and the clustered genes as compared to wild type and to transformants transformed with empty vector. Results are shown in Fig 6. Expression analysis confirmed that expression of the *aflR*-like gene was significantly increased in two independent overexpression transformants as compared to vector-transformed controls. Significantly, cluster genes that were shown to be up-regulated during disease development relative to conidia (Fig 5) were all shown to be up-regulated by overexpressing the *aflR*-like gene; these include *PKS8-1* and the genes encoding the putative glyoxylase/beta-lactamase, cytochrome P450, and the aldo/keto reductase-2. In addition, the gene encoding the MFS transporter was also up-regulated in the overexpression strains. Genes encoding the dehydrogenase and aldo/keto reductase-1, that are not upregulated during disease, were not up-regulated in the *aflR*-like transcription factor overexpressors. Overall, the up-regulation of genes in the cluster by overexpression of the *aflR*-like gene mirrored the expression pattern in the plant relative to conidia, suggesting that overexpression of the *aflR*-like gene was an effective tool in studying the possible role of the pathway in disease. The *aflJ-*like gene was not up-regulated, unlike its expression *in planta*.

### Pathogenicity of *aflR*-like transcription factor overexpressor

**Fig 7:**
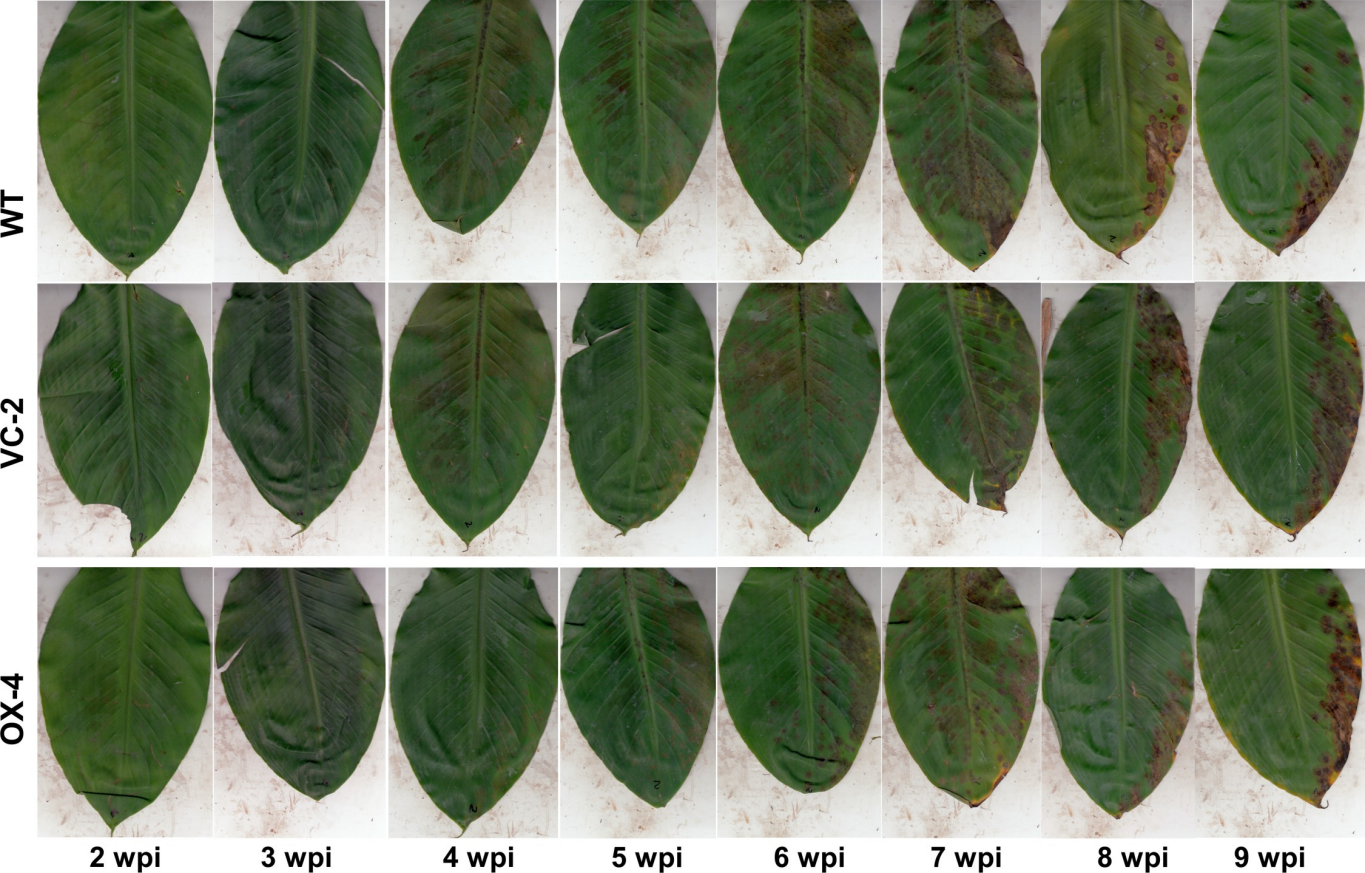
Symptoms on banana following inoculation with the 10CR1-24 wild type, wild type transformed with the vector construct (VC-2) and the overexpression transformant of the *aflR*-like gene (OX-4). Figure shows symptoms on representative leaves from 2 weeks post-inoculation to 9 weeks post-inoculation. There were no differences in disease severity or timing of symptom development between the three treatments.

To determine if overexpression of the *PKS8-1* cluster genes would alter disease development, we conducted inoculation experiments to assay for changes in pathogenicity. Young banana plants were inoculated with conidia, and inoculated leaves were scanned weekly to monitor disease progress over time. Plants were inoculated with the 10CR1-24 wild type, vector control #2 and overexpressor #4. No differences were seen in symptom development or in timing of symptom expression between the three treatments (Fig 7).

### Time course of expression of *PKS8-1* cluster genes in the overexpression mutant *in planta*

**Fig 8:**
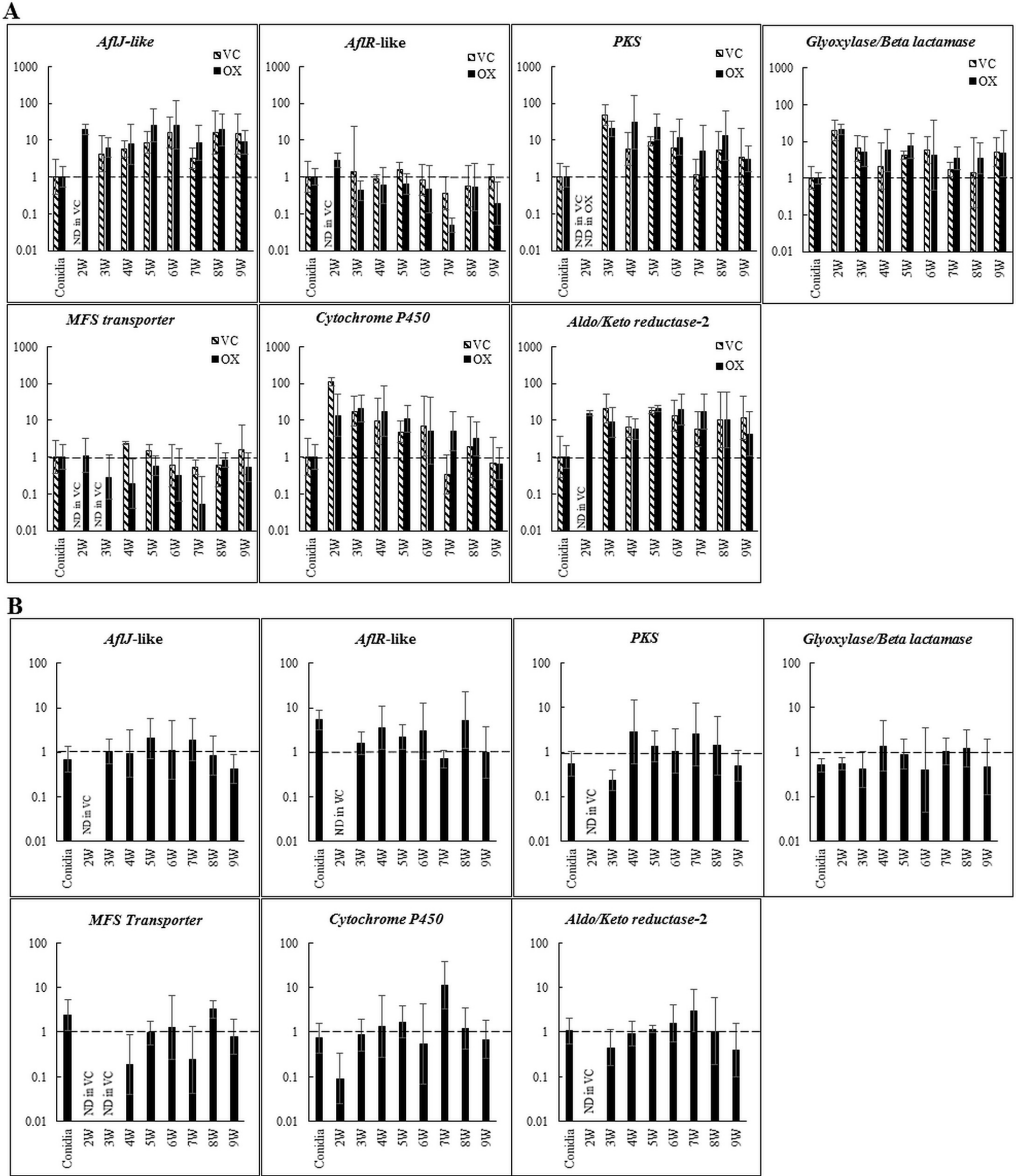
Time course of *PKS8-1* cluster gene expression in the overexpression strain of the *aflR*-like gene during disease development in banana. **A.** Cluster gene expression in the overexpressor and in a vector-transformed control relative to expression in conidia (set to 1) of the respective strains. Black bar = overexpressor strain #4 (OX); hatched bar = vector control #2 (VC). **B.** Same data as in 8A, but cluster gene expression of the overexpressor is shown relative to expression in the vector-control strain (set to 1). Cluster genes assayed are those shown in Fig 4. Tissue was harvested and assayed weekly starting at 2 weeks post inoculation through 9 weeks. Results are shown as a bar graph in log scale. Error bars indicate standard error from three biological replicates. ND = not detected; W = week.

Our results showed that constitutive expression of the *aflR-*like transcription factor gene increased expression of *PKS8-1* and five other cluster genes *in vitro* (Fig 6), however we saw no differences in disease development (Fig 7). To further characterize the impact of *PKS8-1* cluster overexpression during infection and disease we used RT-qPCR to define cluster gene expression *in planta* by an overexpressor and a vector control strain throughout infection and disease development relative to expression in germinating conidia (Fig 8). Overall, the pattern of expression of the cluster genes was the same as with wild type (Fig 5), and there was little or no difference in cluster gene expression *in planta* between the overexpressor and vector-transformed control strain (Fig 8A). Expression of the constitutively expressed *aflR*-like gene was detected earlier than in the control, consistent with overexpression. However, we saw no differences over time in expression of *PKS8-1* between the two strains. Further, expression of the genes that were differentially expressed between the overexpressor and vector control when grown in culture (genes for glyoxylase/beta-lactamase, MFS transporter, cytochrome P450, and aldo/keto reductase-2) (Fig 6) were also not differentially expressed *in planta* (Fig 8A). Fig 8B shows the same data but with expression of the cluster genes in the overexpression transformant calculated relative to the vector control at each time point. We conclude that although overexpression of the *aflR*-like gene is sufficient to increase cluster gene expression in culture, it is not sufficient to increase cluster gene expression *in planta* above the normal *in planta* expression of the cluster. Therefore, it is not possible to make conclusions about the role of the *PKS8-1* cluster in disease development using the overexpression strategy.

## Discussion

Black Sigatoka is an important disease world-wide, yet relatively little is known about pathogenicity factors that the pathogen *P*. *fijiensis* utilizes to cause disease. We have focused studies on polyketide synthases, given their documented importance in production of toxins critically important in disease development by related fungi. Here we show that the *PKS8-1* gene cluster, one of seven PKS gene clusters previously identified in the *P*. *fijiensis* genome [18], is conserved across many Dothideomycete species. Notably, two other closely related pathogens of banana, *P*. *musae* and *P*. *eumusae*, as well as the pine pathogen *P*. *pini-densiflorae*, have orthologous clusters containing an almost identical set of genes. These findings suggest that the cluster encodes a metabolite or metabolites of broad importance to diverse pathogens.

Phylogenetic analysis showed that PKS8-1 forms a clade with the monodictyphenone-producing MdpG PKS from *A*. *nidulans* [38] and the cladofulvin-producing ClaG from *C*. *fulvum* [36, 37]. A comparison of the clustered genes, however, identified only a few shared genes between the three clusters including genes for a beta-lactamase-like enzyme, a decarboxylase, a transcription factor, and a regulatory protein. The *PKS8-1* cluster and the cladofulvin-producing cluster also shared a cytochrome P450-encoding gene. The majority of the genes clustered with *PKS8-1* were not shared with the monodictyphenone or cladofulvin clusters, suggesting production of a different metabolic product. Genes that are shared in the three pathways, however, suggest that *P*. *fijiensis* may synthesize emodin as part of the PKS8-1 pathway. In both *A*. *nidulans* and *C*. *fulvum*, emodin is produced through the activity of the PKS and the proteins encoded by *mpdF* and *mpdH* (in *A*. *nidulans*) and *claF* and *claH* (in *C*. *fulvum*). These proteins are homologous with the glyoxylase/beta-lactamase and the ClaH-like decarboxylase in the *PKS8-1* cluster (Fig 3), suggesting emodin as a possible intermediate in the *P*. *fijiensis* pathway.

RNA-Seq analysis showed that *PKS8-1* and 13 predicted clustered genes were expressed both when the fungus was grown in culture medium and during disease development 6-weeks post-inoculation. However, a time course experiment of expression of cluster genes during disease development showed differential expression *in planta* relative to expression in germinating conidia (Fig 5). The genes encoding PKS8-1, the AflJ-like regulatory protein, the putative glyoxylase/beta lactamase, the cytochrome P-450, and one of the two aldo/keto reductases were all up-regulated in infected leaf tissue relative to expression in germinating conidia. Increased expression could be detected as early as 2-weeks post-inoculation and remained elevated through 9 weeks. Expression of the MFS transporter gene was slightly increased, and was variable during the time course. By contrast, expression of the genes encoding the AflR-like transcription factor, the dehydrogenase, and another aldo/keto reductase were not changed relative to expression in germinating conidia.

To better characterize the cluster, we created an overexpression strain by constitutively expressing the gene encoding the AflR-like transcription factor in the cluster. Expression analysis of cluster genes in two independent overexpression strains confirmed increased expression of the *aflR*-like gene relative to wild type and two vector-transformed control strains. Significantly, other genes up-regulated in culture in the *aflR*-like overexpression strains were the same as those showing increased expression during disease development: genes encoding PKS8-1, glyoxylase/beta lactamase, cytochrome P-450, aldo-keto reductase-2, and the MFS transporter. Genes not up-regulated in infected leaves (encoding the aldo/keto reductase-1 and dehydrogenase) were also not up-regulated in the *aflR*-like overexpression strains. The *aflJ*-like gene was not up-regulated in the overexpression strains, unlike its expression *in planta*. Expression of some but not all clustered genes *in planta* may suggest that the PKS8-1 pathway can generate multiple metabolites, some of which are produced *in planta* and others that are not. It is notable that all of the cluster genes, including those not expressed by *P*. *fijiensis in planta*, are conserved across the homologous clusters in the related fungi *P*. *musae*, *P*. *eumusae*, and *P*. *pini-densiflorae*. We hypothesize that the intermediate generated by the PKS8-1 enzyme may result in production of different metabolites resulting from differential expression of the cluster genes. Production of multiple metabolites encoded by a single gene cluster has been reported in other species. For example, in *A*. *nidulans* the MdpG PKS produces not only monodictyphenone, but also emodin and various emodin analogs [38]. Future work to overexpress the gene encoding the AflJ-like protein may allow for better analysis of the pathway regulation. In *Aspergillus parasiticus* and *Aspergillus flavus*, AflJ has been shown to affect expression of some but not all genes in the aflatoxin pathway [41, 42]. It is possible that the AflJ-like protein in the *PKS8-1* cluster interacts with the *PKS8-1* cluster AflR-like protein to regulate genes encoding various steps of the pathway.

The production of overexpression strains having increased expression of the cluster genes that are up-regulated during disease development also allowed us to test if this cluster plays a role in disease development. No differences were found, however, in symptom expression caused by the overexpression strain relative to wild type and a vector-transformed control. Gene expression analysis showed no differences in *in planta* expression of the cluster genes between the overexpressor compared to the vector-transformed control. We conclude that although overexpression of the *aflR*-like gene is sufficient in culture to increase cluster gene expression, it is not sufficient *in planta* to increase cluster gene expression relative to the normal regulation of the cluster. Thus, it is not possible to make conclusions about the role of the *PKS8-1* cluster in disease development using the overexpression strategy.

It is not clear what role the product(s) of the *PKS8-1* cluster would have in disease. Emodin and emodin derivatives have been shown to have toxicity in animal systems and to some bacteria [37, 43], but there is no information on toxicity to plants. No toxicity was found for cladofulvin in mammalian systems [37], and possible toxicity of monodictyphenone has not been investigated. Further understanding of a possible role of this polyketide pathway in the black Sigatoka disease will await characterization of the metabolite product(s) of the pathway.

## Methods

### RNA-seq analysis of *PKS8-1* gene cluster

Transcriptome sequencing of *P*. *fijiensis* grown in PDB culture medium versus infected banana leaf tissue was previously described [18]. ‘Grand Nain’ banana tissue culture plants were grown in modified Murashige and Skoog medium, and then were transferred to greenhouse conditions in potting mix. Once plants grew to about 20 cm in height, they were transferred to an incubator with an 18 h light/6 h dark photoperiod with cool-white fluorescent lights at 25°C; these plants and PDB flasks were inoculated with conidia of *P*. *fijiensis* isolate 14H1-11A, isolated from Honduras in 2014 [18]. Plants were inoculated by spraying with 20 mL of 5.2 x 10^4^/mL conidia in 0.5% Tween 20, and then were covered with clear plastic bags for 1 week to maintain high humidity conditions. Symptomatic banana tissue at 6 weeks post-inoculation was flash frozen in liquid N_2_ for RNA extraction and transcriptome sequencing. Flasks were inoculated with 10 μL of 1.3×10^6^/mL conidia and incubated in the dark in a rotary shaker at 25–30°C for one week. Mycelium was harvested by filtering through Miracloth and flash freezing in liquid N_2_. Three biological replicates from each condition were analyzed.

The Spectrum Plant Total RNA kit (Sigma) was used for RNA extraction, and samples were treated with DNase I (Roche) to eliminate gDNA. The Genomic Sciences Laboratory (North Carolina State University) conducted total RNA sequencing. An Agilent Bioanalyzer and gel electrophoresis were used to confirm RNA quality, and strand-specific libraries were generated using an NEBNext Ultra Directional library prep kit (NEB). An Illumina HiSeq 2500 instrument was used to generate 125-base single-end reads.

RNA-Seq read quality was verified using the FastQC program (http://www.bioinformatics.babraham.ac.uk/projects/fastqc/), and CutAdapt v1.7 was used to trim Illumina TruSeq adapter sequences and low-quality bases, with a minimum sequence length of 36 and a quality cutoff of 20 [44]. Reads were mapped to both the *P*. *fijiensis* [6, 45] and banana genomes [8, 9] using the program Tophat v2.0.9 [46]. HTSeq v0.6.0 was used to determine gene expression levels [47], and DESeq2 v1.4.5 was used to identify differentially expressed genes [48]. RT-qPCR assays were used with several genes to validate results from the RNA-Seq analysis [19].

### Comparing *P*. *fijiensis* genes in the *PKS8-1* gene cluster with putative orthologs

Tblastn searches were done using BLAST+ [49] for each predicted protein encoded by the *P*. *fijiensis* gene cluster, using the same set of Dothideomycete genomes as was used for a previous analysis [18] (S1 Table). This information was used to determine if *PKS8-1* homologs were flanked by similar genes in other species.

A phylogenetic tree was generated using PKS protein sequences described previously [18], PKS8-1 homologs identified in S1 Table from *P*. *musae* and *P*. *eumusae* (NCBI accessions KXT14292.1 and KXS96182.1, respectively), the cladofulvin-producing PKS ClaG from *C*. *fulvum*, and the monodictyphenone-producing PKS MdpG from *Aspergillus nidulans* (NCBI accession CBF90097.1). PKS protein sequences were aligned using Mesquite v3.51 [50] with MUSCLE v3.8.31 [51]. ModelGenerator v0.85 [52] was used to identify LG+I+G+F [53] as the best evolutionary model, using both the Akaike and Bayesian Information Criteria. RaxmlGUI v1.3.1 [54] was used to generate a maximum likelihood phylogenetic tree with the LG+I+G+F evolutionary model, slow bootstrap, no outgroup, and the autoMRE function.

To compare the *P*. *fijiensis PKS8-1* gene cluster with the *mdpG* gene cluster from *A*. *nidulans* and the *claG* gene cluster from *C*. *fulvum*, blastp searches of each protein sequence encoded by the putative *PKS8-1* gene cluster were performed against the *A*. *nidulans* [55] and *C*. *fulvum* [45] filtered model proteins through JGI. The top hit for each protein sequence was then used for a blastp search against the *P*. *fijiensis* gene catalog protein models [6] to identify any reciprocal best hits. For each protein sequence encoded by the *mdpG* gene cluster from *A*. *nidulans* and the *claG* gene cluster from *C*. *fulvum* as well as two additional genes from *C*. *fulvum* believed to be involved in cladofulvin biosynthesis [37], blastp searches were performed against the *P*. *fijiensis* gene catalog protein models [6] through JGI. The top hit for each protein sequence was used for blastp searches against the *A*. *nidulans* and *C*. *fulvum* filtered model proteins through JGI to identify any reciprocal best hits (Fig 3, S2 Table).

### Creating *aflR*-like transcription factor gene overexpressor

The fungal *trpC* terminator was amplified from pTROYA [56] using primers to add XbaI and HindIII sites. The XbaI and HindIII sites were used to replace the OCS terminator in pEarleyGate 100 [57]. A hygromycin resistance cassette was amplified from plasmid pCB1636 [58] with primers to add HindIII restriction sites to both ends of the PCR product. The HindIII site in the modified pEarleyGate 100 vector was then used to insert the hygromycin resistance cassette as a fungal selectable marker. The *trpC* promoter was amplified from pTROYA with primers to add SacI and XhoI sites. The SacI and XhoI sites in the modified pEarleyGate 100 were used to replace the 35S:Bar plant selectable marker with a fungal *trpC* promoter, to create the Gateway destination vector. The *aflR*-like transcription factor gene was amplified from isolate 96CAM275 (isolated by Ronald Romero in Cameroon and kindly provided by Turner Sutton, North Carolina State University), and inserted into pDONR221 via Gateway BP clonase (Life Technologies) reaction to create entry clones containing the transcription factor sequence. Finally, the sequence was moved into the destination vector using Gateway LR clonase (Life Technologies) to create the expression vector. To create a hygromycin cassette-only control, promoterless GFP with a trpC terminator was amplified from the vector pRG2 (kindly provided by G. A. Payne, North Carolina State University) using the primers 5’-ACGGTAACTAGTGCTTGAGCAGACATCACC-3’ and 5’-TTAATTAAGATTAAGTTGGGTAACGCCA-3’. The PCR product was digested with HindIII and SpeI, and inserted into pEarleyGate 100 [57] using the compatible HindIII and XbaI sites. The hygromycin resistance cassette was amplified from plasmid pCB1636 [58] using primers 5’-CGACTGAAGCTTTCGACGTTAACTGGTTCCC-3’ and 5'-GCATATAAGCTTCGTTAACTGATATTGAAGGAGCA-3’ to add HindIII restriction sites. The PCR product was digested with HindIII, and then was inserted into the modified pEarleyGate 100 vector. The modified pEarleyGate 100 with promoterless GFP and a hygromycin resistance cassette was digested with EcoRI and XbaI to remove the GFP sequence. The vector was treated with Klenow enzyme to generate blunt ends, and was ligated back together to generate a modified pEarleyGate 100 with a hygromycin resistance cassette in the T-DNA (S1 Fig).

*P*. *fijiensis* was transformed using *A*. *tumefaciens* strain EHA105, based on the protocol by Utermark and Karlovsky [59]. Briefly, EHA105 with the appropriate plasmid was grown in liquid Lysogeny Broth (LB) medium with 50 μg/mL each kanamycin and rifampicin to an OD_600_ of 0.5 to 0.9, washed in Induction Medium (IM) and resuspended in IM supplemented with 200 μM acetosyringone. Cells were then grown to an OD_600_ of 0.3, mixed with *P*. *fijiensis* conidia, and spread onto cellophane covering solid IM plates. Plates were incubated for one week at room temperature, and then cellophane was transferred to PDA with 125 mg/L hygromycin and 0.56 g/L ticarcillin, with additional PDA with hygromycin and ticarcillin poured on top of the cellophane to select for transformants and to kill the *A*. *tumefaciens*. After about 3 weeks, colonies appeared and were transferred to new plates for further analysis.

### Banana inoculations

Banana tissue culture plants of the Grand Nain cultivar were kindly provided by Miguel Muñoz, Dole Food Company, and were maintained on modified Murashige and Skoog medium as previously described, on an 18h light/6h dark photoperiod with cool-white fluorescent lights at 25–30°C [18]. Rooted *in-vitro* cultured banana plants were transferred to potting mix and grown in the North Carolina State University Phytotron facility with greenhouse conditions on a 12h light/12h dark photoperiod at 26°C and 22°C in the day and night, respectively. *P*. *fijiensis* conidia were produced and harvested as previously described [18], and 5 mL of 2x10^4^ conidia/mL were atomized onto young banana plants (about 20 cm in height). Three independent inoculation experiments were performed, with at least eight plants inoculated per fungal genotype. For one-week post-inoculation, each plant was covered in a clear plastic bag to maintain high humidity conditions. Plants were harvested for expression analysis by flash freezing symptomatic leaf tissue in liquid nitrogen. Inoculated leaves were scanned using a CanoScan LiDE 220 scanner.

### Expression assays of genes in the *PKS8-1* gene cluster

Total RNA was isolated using a Spectrum Plant Total RNA kit (Sigma), treated with recombinant RNase-free DNase I (Roche) to eliminate gDNA, and reverse transcribed using iScript Select cDNA synthesis kit (Bio-Rad), according to manufacturer’s instructions. In order to obtain an unbiased target-specific preamplification of the limited amounts of fungal nucleic acid transcripts, cDNA was preamplified using SsoAdvanced PreAmp Supermix (Bio-Rad). The primers for this custom-designed assay were comprised of 50 nM of each primer for each of the *PKS8-1* cluster genes along with the reference genes. The thermal cycling protocol involved an initial denaturation step of 95°C for 3 minutes, followed by 12 cycles of 95°C for 15 seconds and an annealing step of 58°C for 4 minutes. After diluting each preamplified cDNA sample to a ratio of 1:5 with Low EDTA TE buffer (USB Corp.), qPCR was set up with iQ SYBR Green Supermix (Bio-Rad). The thermal cycling protocol included an initial denaturation of 95°C for 2 minutes, followed by 45 cycles of 95°C for 10 seconds, target-dependent annealing temperature for 30 seconds, and 72°C for 30 seconds with a plate read. Melt curves were used to verify amplification of a single product for each reaction. Primer sequences are indicated in S3 Table. Each gene of interest was normalized against two *P*. *fijiensis* reference genes having the same efficiency as the gene of interest, and fold-change was calculated using the 2^−ΔΔC^_T_ method [60].

## Supporting information

S1 Fig Cloning plasmids for transformation of *P*. *fijiensis*. The OCS terminator from vector pEarleyGate 100 [57] (A) was removed using the XbaI and HindIII sites, and was replaced with a PCR product amplified from pTROYA [56] of the fungal *trpC* terminator, to create (B). A hygromycin resistance cassette was amplified from the plasmid pCB1636 [58], and was inserted into the modified pEarleyGate vector (B) using the HindIII site, to create (C). A fungal *trpC* promoter was amplified from pTROYA [56] and was used to replace the 35S:Bar cassette in (C), using the SacI and XhoI sites, to create the destination vector (D). The *aflR*-like transcription factor gene was amplified from *P*. *fijiensis* and was moved into the plasmid pDONR221 via a Gateway BP reaction to create the entry vector (E), and a Gateway LR reaction was used to generate the expression vector (F). To generate the hygromycin resistance cassette-only vector control, promoterless GFP was amplified from the vector pRG2 (kindly provided by G. A. Payne, North Carolina State University) and inserted into pEarleyGate 100 using the HindIII and EcoRI sites. Then the Hph selectable marker was amplified from pCB1636 and inserted using the HindIII site, generating a modified pEarleyGate 100 with a promoterless GFP sequence and a hygromycin resistance cassette (G). This vector was digested with EcoRI and XbaI, treated with Klenow enzyme, and ligated back together to create the hygromycin resistance cassette-only control vector (H).
(TIF)

S1 Table Homologs of putative *PKS8-1* biosynthetic cluster genes in selected Dothideomycetes. For each gene in the putative *PKS8-1* biosynthetic cluster from *P*. *fijiensis*, tblastn searches were done using BLAST+ with the Dothideomycete genomes listed previously [18]. The top 25 hits by bitscore are shown in the table, with hits from *P*. *eumusae*, *P*. *musae*, and *P*. *pini-densiflorae* shown in red. The table indicates the species and genome scaffold from which the hit was found, the percent protein identity and similarity, the bitscore, the E-value, the number of gap openings (Gap open), the number of mismatches (Mismatch), the alignment length (Length), the start and end of the alignment in query (Qstart and Qend), and the start and end of the alignment in subject (Sstart and Send). Each tab shows the hits for a different gene in the putative *PKS8-1* biosynthetic cluster. A) AflJ-like protein (accession XP_007929877.1); B) Hypothetical protein (accession XP_007929878.1); C) Hypothetical protein (accession XP_007929879.1); D) AflR-like transcription factor (accession XP_007929880.1); E) Hypothetical protein (accession XP_007929881.1); F) Hypothetical protein (accession XP_007929882.1); G) PKS8-1 (accession XP_007929626.1); H) Glyoxylase/beta-lactamase-like protein (accession XP_007929883.1); I) Hypothetical protein (accession XP_007929629.1); J) ClaH-like decarboxylase (accession XP_007929611.1); K) MFS transporter (accession XP_007929884.1); L) Cytochrome P450 (accession XP_007929885.1); M) Hypothetical protein (accession XP_007929886.1); N) Aldo/keto reductase (accession XP_007929887.1); O) Dehydrogenase/ 3-ketoacyl-(acyl-carrier-protein) reductase (accession XP_007929584.1); P) Aldo/keto reductase (accession XP_007929888.1); Q) Transcription factor (accession XP_007929889.1).
(XLSX)

S2 Table Reciprocal blast hits of the *P*. *fijiensis* putative *PKS8-1* biosynthetic cluster compared to the putative monodictyphenone and cladofulvin-producing clusters in *A*. *nidulans* and *C*. *fulvum*, respectively. Reciprocal blast hits in the putative *PKS8-1* biosynthetic cluster compared to the monodictyphenone-producing and the cladofulvin-producing clusters were identified via blastp searches using the respective genomes available on JGI [6, 45, 55]. For each query sequence, the putative function and accession or JGI protein ID are indicated, as well as the protein length. For each of the best blastp hits, the JGI protein ID, the genome scaffold and position, the gene and protein length, the E-value, the percent identity, and the percent subject coverage are indicated. Red text indicates reciprocal best hits for which the subject sequence is clustered with the PKS or is believed to be involved in the biosynthetic pathway [37]. Blue text indicates reciprocal best hits for which the subject sequence is neither clustered with the PKS nor believed to be involved in the biosynthetic pathway [37]. Black text indicates a lack of reciprocal best hits. Each tab shows the results for a different gene cluster comparison. A) For each of the genes neighboring *PKS8-1* in the *P*. *fijiensis* genome, the best hit is shown in the *C*. *fulvum* genome; B) For each of the genes neighboring the PKS *claG* gene in the *C*. *fulvum* genome, as well as for additional genes proposed to be involved in cladofulvin biosynthesis [37], the best hit is shown in the *P*. *fijiensis* genome; C) For each of the genes neighboring *PKS8-1* in the *P*. *fijiensis* genome, the best hit is shown in the *A*. *nidulans* genome; D) For each of the genes neighboring the PKS *mdpG* in the *A*. *nidulans* genome, the best hit is shown in the *P*. *fijiensis* genome.
(XLSX)

S3 Table Primer sequences and reference genes used for RT-qPCR assays. For each gene assayed in RT-qPCR assays from Figs 5, 6 and 8, the table indicates the gene assayed, annealing temperature of the primers, the forward and reverse primer sequences, two reference genes for each gene of interest, and amplicon sizes.
(XLSX)
