# Supplementary figures and images for: A novel polyketide synthase gene cluster in the plant pathogenic fungus Pseudocercospora fijiensis
Source: PLoS One. 2019 Feb 8;14(2):e0212229. doi: 10.1371/journal.pone.0212229 (PMC6368318; doi:10.1371/journal.pone.0212229)

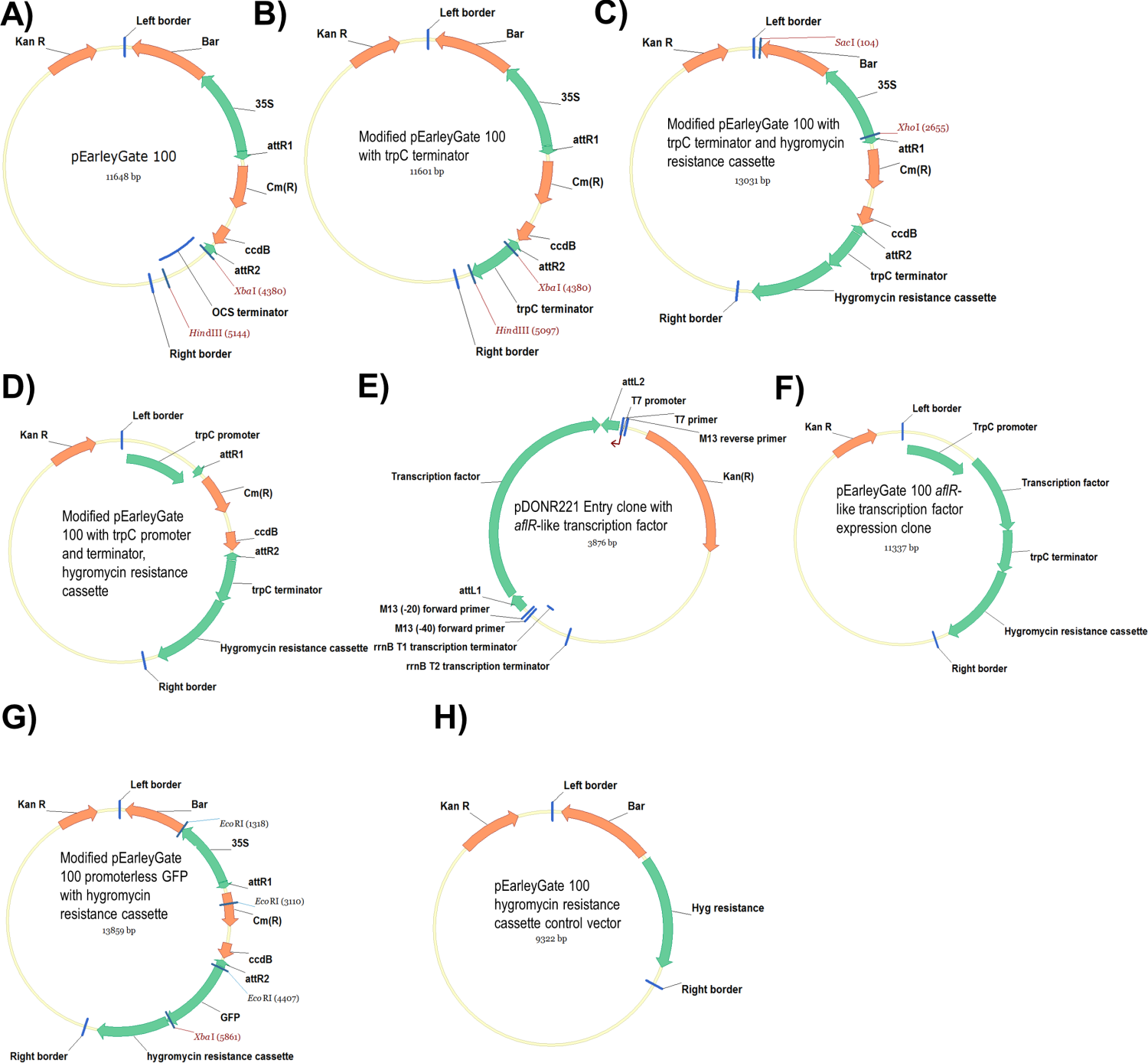

Supplement: S1 Fig — The OCS terminator from vector pEarleyGate 100 [57] (A) was removed using the XbaI and HindIII sites, and was replaced with a PCR product amplified from pTROYA [56] of the fungal trpC terminator, to create (B). A hygromycin resistance cassette was amplified from the plasmid pCB1636 [58], and was inserted into the modified pEarleyGate vector (B) using the HindIII site, to create (C). A fungal trpC promoter was amplified from pTROYA [56] and was used to replace the 35S:Bar cassette in (C), using the SacI and XhoI sites, to create the destination vector (D). The aflR-like transcription factor gene was amplified from P. fijiensis and was moved into the plasmid pDONR221 via a Gateway BP reaction to create the entry vector (E), and a Gateway LR reaction was used to generate the expression vector (F). To generate the hygromycin resistance cassette-only vector control, promoterless GFP was amplified from the vector pRG2 (kindly provided by G. A. Payne, North Carolina State University) and inserted into pEarleyGate 100 using the HindIII and EcoRI sites. Then the Hph selectable marker was amplified from pCB1636 and inserted using the HindIII site, generating a modified pEarleyGate 100 with a promoterless GFP sequence and a hygromycin resistance cassette (G). This vector was digested with EcoRI and XbaI, treated with Klenow enzyme, and ligated back together to create the hygromycin resistance cassette-only control vector (H). (TIF) [file pone.0212229.s001.tif]
